# Kansas State Dental Laws

**Published:** 1885-03

**Authors:** 


ARTICLE V.

### KANSAS STATE DENTAL LAWS.

### AN ACT.

To regulate the practice of dentistry, and punish violators thereof.

*Be it enacted by the Legislature of the State of Kansas:*

Sec. I. That it shall be unlawful for any person to practice or attempt to practice dentistry or dental surgery in the State of Kansas, without first having received a diploma from the faculty of some reputable Dental College, School, or University Department, duly authorized by the laws of this State, or some other of the United States, or by the laws of some foreign government, and in which College, School, or University Department, there was at the time of the issuance of such diploma, annually delivered a full course of lectures and instructions in dentistry or dental surgery.

*Provided,* That nothing in Sec. I of this Act shall apply to any person engaged in the practice of dentistry or dental surgery in this State at the time of the passage of this Act, except as hereinafter provided.

*And, provided further,* That nothing in this Act shall be so construed as to prevent Physicians, Surgeons or others from extracting teeth.

Sec. II. A Board of Examiners, consisting of five practicing dentists, residents of this State, is hereby created, who shall have authority to issue certificates to persons in the practice of dentistry or dental surgery in the State at the time of the passage of this Act, and also to decide upon the validity of such diplomas as may be subsequently presented for registration, as hereinafter provided.

Sec. III. The members of said Board shall be appointed by the Governor, and shall serve for a term of four years, excepting that the members of the Board first appointed shall hold their offices as follows: Two for two, and three for four years respectively, and until their successors are duly appointed. In case of vacancy occuring in said Board, such vacancy shall be filled by appointment by the Governor.

Sec. IV. Said Board shall keep a record, in which shall be registered the names and residence or place of business of all persons authorized under this Act to practice dentistry or dental surgery in this State. It shall elect one of its members President, and one Secretary thereof, and it shall meet at least once in each year, and as much oftener, and at such times and places as it may deem necessary. A majority of the members of said Board shall constitute a quorum, and the proceedings thereof shall be at all times open for public inspection.

Sec. V. Every person engaged in the practice of dentistry or dental surgery within this State at the time of the passage of this Act, shall within six months thereafter cause his or her name and residence and place of business to be registered with said Board of Examiners, upon which said Board shall issue to such person a certificate duly signed by a majority of the members of said Board, and which certificate shall entitle the person to whom it is issued to all the rights and privileges set forth in Sec. 1 of this Act.

Sec. VI. Any person desiring to commence the practice of dentistry or dental surgery within this State after the passage of this Act, shall, before commencing such practice, file for record in a book kept for such purpose, with said Board of Examiners, his or her diploma, or a duly authenticated copy thereof, the validity of which said Board shall have power to determine. If accepted, said Board shall issue to the person holding such diploma a certificate duly signed by all or a majority of the members of said Board, and which certificate shall entitle the person to whom it is issued to all the rights and privileges set forth in Sec. 1 of this Act.

Sec. VII. To provide for the proper and effective enforcement of this Act, said Board of Examiners shall be entitled to the following fees, TO-WIT: For each certificate issued to persons engaged in practice in this State at the time of the passage of this Act, the sum of Three Dollars. For each certificate issued to the persons not engaged in the practice of dentistry at the time of the passage of this Act, the sum of Ten Dollars.

Sec. VIII. The members of said Board shall each receive the compensation of three dollars per day for each day actually engaged in the duties of their office, which, together with all other legitimate expenses incurred in the performance of such duties, shall be paid from fees received by the Board under the provisions of this Act, and no part of the expenses of said Board shall at any time be paid out of the State treasury.

All monies in excess of said *per diem* allowance, and other expenses, shall be held by the Secretary of said Board as a special fund for meeting the expenses of said Board, he giving such bond as the Board shall from time to time direct, and such Board shall make an annual report of its proceedings to the Governor by the fifteenth day of December of each year, together with an account of all monies received and disbursed by them in pursuance of this Act.

Sec. IX, Any person who shall violate this Act by practicing or attempting to practice dentistry within the State without first complying with the provisions of this Act, shall be deemed guilty of a misdemeanor, and upon conviction thereof shall be fined in a sum not less than Ten Dollars nor more than one hundred dollars for each offense. All fines recovered under this Act shall be paid into the common school fund of the county in which the conviction is obtained.

Sec. X. This Act shall take effect and be in force from and after publication in the Statutes.

